# In Situ Synthesis of Alumina Nanoparticles in a Binary Carbonate Salt Eutectic for Enhancing Heat Capacity

**DOI:** 10.3390/nano10112131

**Published:** 2020-10-27

**Authors:** Yousof Nayfeh, Syed Muhammad Mujtaba Rizvi, Baha El Far, Donghyun Shin

**Affiliations:** School of Engineering and Technology, Central Michigan University, Mt Pleasant, MI 48859, USA; nayfe1y@cmich.edu (Y.N.); rizvi1sm@cmich.edu (S.M.M.R.); elfar1b@cmich.edu (B.E.F.)

**Keywords:** nanomaterial, thermal energy storage, molten salt, specific heat, in situ synthesis

## Abstract

A binary carbonate salt eutectic (Li_2_CO_3_-K_2_CO_3_)-based nanofluid was in situ synthesized by mixing with a precursor material, aluminum nitrate nonahydrate (Al(NO_3_)_3_·9H_2_O). Thermal decomposition of the precursor was successfully carried out to synthesize alumina (Al_2_O_3_) nanoparticles at 1 wt.% concentration. A thermogravimetric analysis (TGA) confirmed a complete thermal decomposition of aluminum nitrate nonahydrate to alumina nanoparticles. A transmission electron microscope (TEM) was employed to confirm the size and shape of the in situ formed nanoparticles; the result showed that they are spherical in shape and the average size was 28.7 nm with a standard deviation of 11.7 nm. Electron dispersive X-ray spectroscopy (EDS) confirmed the observed nanoparticles are alumina nanoparticles. A scanning electron microscope (SEM) was employed to study microstructural changes in the salt. A differential scanning calorimeter (DSC) was employed to study the heat capacity of the in situ synthesized nanofluid. The result showed that the heat capacity was enhanced by 21% at 550 °C in comparison with pure carbonate salt eutectic. About 10–11 °C decrease of the onset melting point of the binary carbonate salt eutectic was observed for the in situ synthesized nanofluids.

## 1. Introduction

Molten salts such as nitrate salts, carbonate salts, chloride salts, and their mixtures are known for promising thermal energy storage media at high-temperature applications. Enhancing the heat capacity of molten salts can significantly improve their performance as thermal energy storage because it can not only reduce the material to store heat but also minimize associated heat transfer systems (e.g., tanks, pipes, heat exchangers, etc.). Therefore, many researchers have worked on enhancing the thermal properties of molten salts and their mixtures [1]. Nanofluids are fluids doped with nanoparticles. They are known for their large enhancement in thermal conductivity [2]. A number of experimental studies have been reported to show largely enhanced thermal conductivity values of various nanofluids [2,3,4,5]. Similarly, the heat capacity of molten salts can be also enhanced by doping with nanoparticles. Since heat capacity enhancements were first reported for carbonate salt-based and chloride salt-based nanofluids in 2011, many studies have reported showing enhanced heat capacity of various molten salt-based nanofluids such as nitrate salts [6], carbonate salts [7], and chloride salts [8]. Several studies have been proposed to explain the underlying mechanism behind the experimentally observed heat capacity enhancements. They include enhanced heat capacity of nanoparticles over bulk materials [9], interfacial thermal resistance as secondary heat storage [10], semi-solid liquid layering of salt molecules at the surface of nanoparticles [11], ionic exchange heat capacity of nanoparticles, and nano-dendritic formation of salts near nanoparticles [12].

Various synthesis techniques were also studied for enhancing the heat capacity of molten salt nanofluids. One of the most commonly used synthesis techniques is the liquid solution method first reported in 2011 [8]. Salts are mixed with nanoparticles and dissolved in distilled water. The solution is sonicated to disperse nanoparticles homogeneously, and then the water is evaporated by heating the solution on a hot plate or inside a furnace. The sonication step helps nanoparticles disperse well in the solution; however, nanoparticle aggregation can occur during the time-consuming evaporation step, which can take up to 200 min. An additional refining step was reported later to improve the liquid solution method [13]; however, the refining step could result in spatial non-homogeneity mixing of molten salts. Other proposed production methods include magnetic or mechanical stirring of the salt and nanoparticle mixture at high temperatures in the liquid phase to disperse the nanoparticles [14,15,16], and mechanical dry mixing using a ball mill with stainless steel bearings [17]. Recently, Lasfargues et al. [18] proposed a one-step method using a precursor material to create nanoparticles instead of dispersing commercial nanoparticles. This method can reduce potential nanoparticle aggregations by eliminating the evaporation step in the liquid solution method. It can not only reduce the manufacturing cost by simplifying the synthesis procedure, but also reduce the material cost because it does not need commercial nanoparticles. In this method, a precursor material is mixed well with salt mixture, and the whole mixture is heated in a furnace for several hours to thermally decompose the precursor material into nanoparticles. However, there are limited literature available about the in situ synthesis method and all the reported studies focused only on nitrate salt-based nanofluids [18,19,20,21].

Hence, in this paper, we investigated selected precursor material for carbonate salt-based nanofluids. Alumina (Al_2_O_3_) nanoparticles (at 1 wt.% concentration) were in situ synthesized in a binary eutectic mixture of lithium carbonate and potassium carbonate (62:38 by molar ratio). This carbonate salt eutectic is stable up to very high temperatures and reported for their large enhancement in heat capacity after being mixed with nanoparticles [22,23]. A differential scanning calorimeter (DSC) and a thermogravimetric analysis (TGA) were employed to study the specific heat capacity and thermal stability of nanofluids. A transmission electron microscope (TEM) and energy-dispersive X-ray spectroscopy (EDS) were employed to confirm the formation of alumina nanoparticles. A scanning electron microscope (SEM) was used to investigate micro-structural changes in the nanofluids.

## 2. Method

Lithium carbonate (Li_2_CO_3_, 99%), potassium carbonate (K_2_CO_3_, 99%), and aluminum nitrate nonahydrate (Al(NO_3_)_3_·9H_2_O, 98%) were procured by Alfa Aesar (Haverhill, MA, USA), Fisher Chemical (Waltham, MA, USA), and Sigma Aldrich (St. Louis, MO, USA), respectively. Around 460.60 mg of lithium carbonate and 529.40 mg of potassium carbonate were measured precisely using a microbalance (SECURA 225D, Sartorius, Göttingen, Germany) and mixed to prepare 990.00 mg of Li_2_CO_3_-K_2_CO_3_ eutectic (62:38 by molar ratio). A total of 73.56 mg of aluminum nitrate nonahydrate powder (i.e., precursor powder) was precisely measured and mixed with the eutectic of Li_2_CO_3_-K_2_CO_3_. The total of 73.56 mg was calculated to yield a theoretical concentration of 1 wt.% of alumina nanoparticles in the salt eutectic. The process of geometric dilution was used to disperse the precursor powder in the salt eutectic homogenously. An equal amount of the salt eutectic and the precursor powder was mixed and ground using a pestle and mortar for 15 min until the mixture became a fine powder. Then the same amount of the salt eutectic as the amount of the original precursor powder was added to the pestle and ground. This is a widely used method in the pharmaceutical field to mix a small amount of powder into another to ensure a uniform and homogenous mixture [24]. The mixture was then poured into a porcelain crucible and placed inside a laboratory furnace (Naytech Vulcan D-550, Cole-Parmer, Vernon Hills, IL, USA) to heat the mixture up to 560 °C at a heating rate of 30 °C/min (i.e., the maximum allowed by the furnace) and maintained at 560 °C for 3 h. Pure salt eutectic was also prepared in the same process for comparison. The synthesis parameters were designed to achieve smaller particles with good stability since multiple studies were reported that higher heating rates result in smaller and less agglomerated nanoparticles [25,26,27,28,29]. Hong et al. [30] proposed that, at high heating rates, the rate of nucleation is greater than the rate of growth; therefore, nanometer-sized particles are produced. Saita and Maenosono [31] reported that increasing the time in the last step of the thermal decomposition process results in a more uniform nanoparticle size distribution.

A differential scanning calorimeter (DSC; DSC 25, TA Instruments, New Castle, DE, USA) was employed to measure the specific heat capacity of the molten salt nanofluid. A sample of 9–13 mg of the salt was extracted from the crucible and transferred into a Tzero hermetic pan (TA Instruments, New Castle, DE, USA), the pan was then heated on a hot plate (Isotemp Hotplate, Fisher Scientific, Waltham, MA, USA) at 500 °C for 5 min to remove any remaining moisture and then hermetically sealed with a lid (TA Instruments, New Castle, DE, USA). Each sample was heated inside the DSC at a slow heating rate of 2 °C/min starting from 200 °C up to 560 °C and cooled back to 200 °C, this step is known to promote higher heat capacity enhancements in molten salt nanofluids by inducing nano-structural changes in salt mixtures [32]. Then, the specific heat measurement took place in the DSC at a heating rate of 20 °C according to the standard heat capacity testing protocol (ASTM E-1269 [33]). The specific heat capacity measurement was repeated three times to ensure good repeatability of the measurement.

Thermogravimetric analysis (TGA; TGA 55, TA Instruments, New Castle, DE, USA) was carried out to monitor the weight change of each sample during the thermal decomposition process that induces the nanoparticle formation. A sample of about 15 mg was heated to 50 °C and held in an isothermal state for 5 min and heated to 560 °C at a rate of 30 °C/min. The same TGA test was also completed for pure salt eutectic for comparison. Both DSC and TGA were installed in a cleanroom facility to prevent any sample contamination from airborne particles.

The nanoparticles were characterized using a transmission electron microscope (TEM; HT 7700, Hitachi, Tokyo, Japan). The nanofluid was dissolved in water and sonicated in a water bath (5200 ultrasonic cleaner, Branson, Brookfield, CT, USA) for two hours to remove the salt eutectic so that nanoparticles can be seen. A small sample was taken using a pipette and dropped onto a carbon-coated 400 mesh copper grid (Electron Microscopy Sciences, Hatfield, PA, USA) and dried by air. Then, a droplet of distilled water was dropped onto the grid’s surface and wicked off using a filter paper multiple times to remove any remaining salts. Beam alignment and stigmation were adjusted at a magnification of ×50,000. A voltage of 100 kV was applied between the cathode and anode. All images were taken in the high-resolution mode to better analyze the size and shape of the nanoparticles. Moreover, electron dispersive X-ray spectroscopy (EDS) was used to confirm the presence of alumina nanoparticles.

A scanning electron microscope (SEM; 3400N-II, Hitachi) was used to study the micro/nanostructure of the salt after being doped with nanoparticles to observe any changes in the structure since the thermophysical properties of any material is usually linked to its phase and structure [12,34,35,36,37]. The samples were mounted on aluminum stubs covered with conductive double-sided carbon tape. The accelerating voltage used was between 10 and 15 kV, and the objective aperture was between 30 and 50 μm.

## 3. Results and Discussion

### 3.1. Thermogravimetric Analysis (TGA)

**Figure 1:**
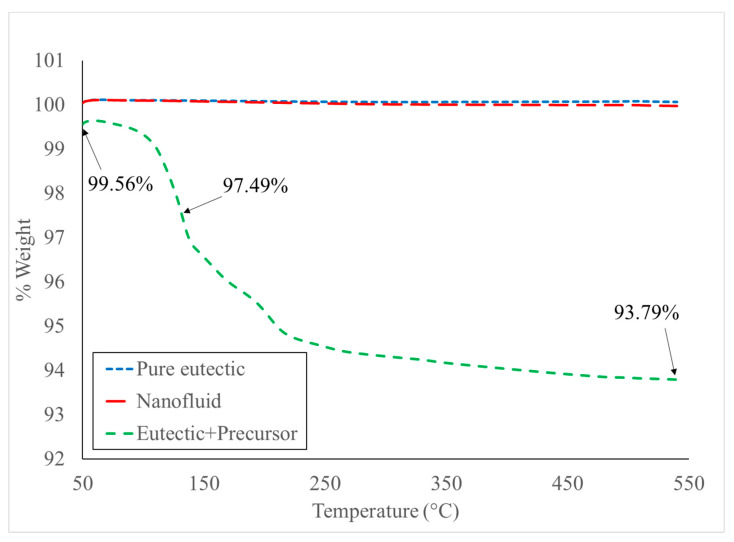
TGA curves of the pure salt eutectic, its mixture with alumina precursor (6.92 wt.%), during the thermal decomposition, and the final nanofluid.

The TGA curve of the salt with aluminum nitrate nonahydrate (6.92 wt.%) is shown in Figure 1. The chemical formula that describes the thermal decomposition of aluminum nitrate nonahydrate proposed by El-Shereafy et al. [38] is as follows:

(1) $$2Al{\left(NO_{3}\right)}_{3}\cdot 9H_{2}O\underset{130\;{}^{\circ}C}{\to }2Al\left(NO_{3}\right)+9H_{2}O\underset{200\;{}^{\circ}C}{\to }Al_{2}O_{3}+3NO_{2}+3NO+3O_{2}+9H_{2}O$$

Based on Equation (1), the weight percentage of the final product after a complete thermal decomposition is 94.02% as summarized in Table 1. The experimentally measured weight percentage (by TGA) of the nanofluid at 540 °C was 93.79%. The result shows that a complete thermal decomposition of the aluminum nitrate nonahydrate occurred at least by 540 °C. The TGA curve shown in Figure 1 is not completely plateaued at this point; therefore, the sample was kept in the furnace at 540 °C for an additional 3 h to ensure that the decomposition process is completed. In addition, Figure 1 also includes the TGA results of a pure binary carbonate salt eutectic, and no significant mass loss or decomposition was observed.

**Figure 2:**
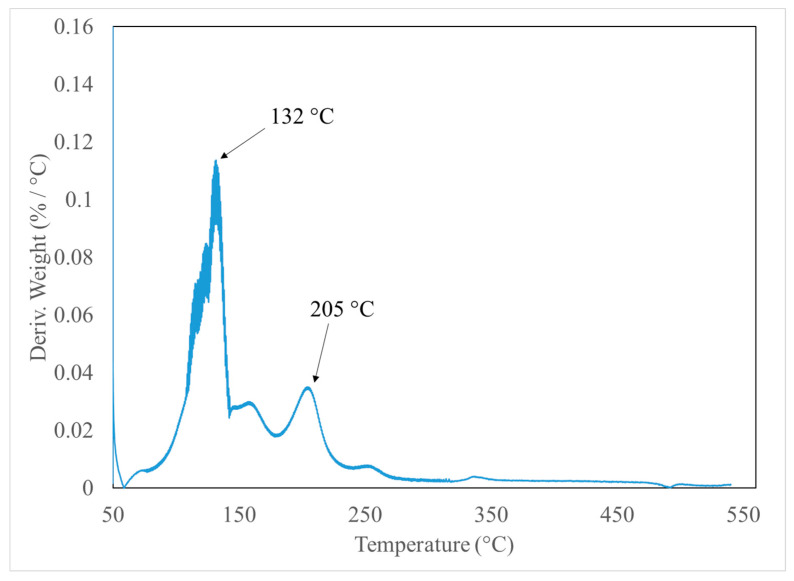
A derivative of the TGA curve representing the rate of mass loss with temperature.

**Figure 3:**
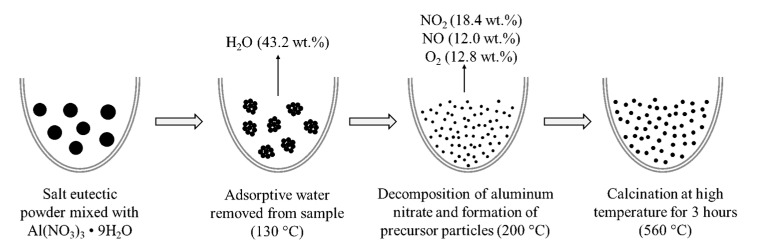
A schematic of the in situ alumina nanoparticle formation via thermal decomposition process.

Moreover, El-Shereafy et al. [38] reported that Al(NO_3_)_3_·9H_2_O shows a strong endothermic peak at 130 °C due to the loss of the adsorptive water molecules, and the second step of the decomposition takes place at 200 °C as shown in Equation (1). The TGA result agreed well with the literature. Two peaks were observed at 132 °C and 205 °C on the derivative of the TGA curve shown in Figure 2. The peak at 132 °C is due to the hydrate adsorptive water molecules. It is about 43% of Al(NO_3_)_3_·9H_2_O and about 3% of the total mass of the nanofluid. The weight percentage at 132 °C was 97.49% and reached 97.01% at 137 °C. A smaller peak was observed at 205 °C corresponding to the temperature at which the thermal decomposition of aluminum nitrate starts. Lee et al. [39] successfully prepared alumina nanoparticles via calcination of aluminum nitrate nonahydrate. They reported that the alumina precursor particles formed around 180 °C when the color of the sample changed to dark brown. The same color change was observed in our sample during heating as well and is due to the nitrogen dioxide emitted during the thermal decomposition of Al(NO_3_)_3_·9H_2_O. Oskam [40] reported that there is a growth phase after the formation and nucleation of the precursor during the synthesis of metal oxide nanoparticles. The subsequent phases are coarsening and aggregation. They explained that nanoparticles will grow spherical because it represents the shape with the lowest surface energy. However, some particles may aggregate randomly and weld together. To reduce possible agglomeration, the samples were left in the furnace for an additional three hours at the end of the decomposition process at 560 °C. According to Saita and Maenosono [31], it helps achieve a uniform size distribution of the nanoparticles. Figure 3 summarizes each step of the in situ nanoparticle formation.

### 3.2. DSC Measurements

Table 2 shows a summary of the DSC measurements for three pure salt eutectic (Li_2_CO_3_-K_2_CO_3_) samples prepared and tested on different days from different batches. Each sample was repeated three times in the DSC to show the repeatability of the measurement. The average heat capacity values and the standard deviation of each sample across the three measurements are reported at 550 °C (Table 2). The average specific heat capacity (C_p_) value was 1.61 kJ/kg·°C which made a good agreement with the literature value [7,22]. The measurement uncertainty was calculated by *t*_0.95,*k*_ × *S_x_/x*. *t*_0.95,*k*_ is Student *t*-value for 0.95 confidence probability. *k* is the degree of freedom, *k* = *n* − 1. *n* is the number of measurements. *S_x_* is the variance and *x* is the average value. The random uncertainty value of the pure salt eutectic (9 measurements) was only 3.31%.

**Figure 4:**
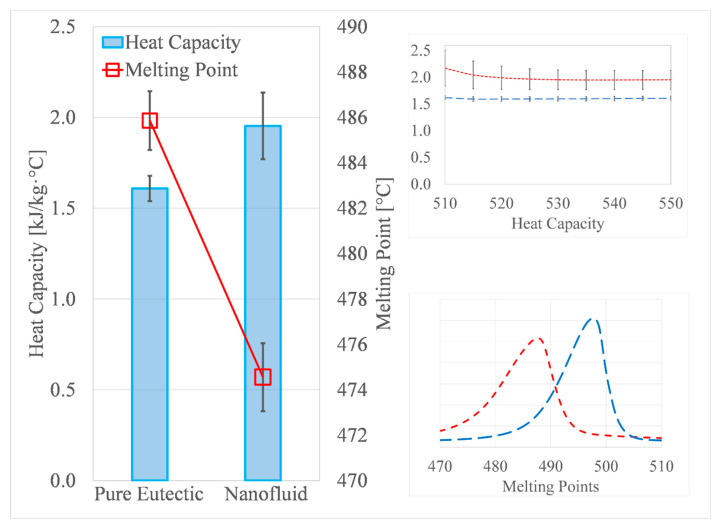
The DSC curves of the pure salt eutectic and its nanofluid. The error bars represent the standard deviation.

Twelve samples of the nanofluid were synthesized and tested by the DSC on different days. Each sample was repeated three times to show the measurement repeatability. The result is available in Table 3. The average specific heat capacity value was 1.95 kJ/kg·°C and the measurement uncertainty for all 36 measurements was 3.18%. The enhancement corresponds to an average enhancement of 21.1%. The value is close to C_p_ reported by other studies on the same base salt (Li_2_CO_3_-K_2_CO_3_) using the conventional liquid solution method [7,41,42]. Figure 4 shows the average DSC curves of all the samples reported of both pure and nanofluid salts.

Moreover, the effect of the addition of nanoparticles on the melting point of the salt was investigated as shown in Table 2 and Table 3. The onset melting point of each sample was calculated by the point of intersection of the linearly extrapolated baseline of the DSC heat flow curve with the tangent line of the maximum slope which goes through the peak [43,44]. It was found that the melting point of the pure salt eutectic mixture ranges between 485 and 487 °C, which is in line with the values reported by other researchers [22,41,42]. The salt nanofluid showed a decrease in melting point across all samples as the value ranged between 471 and 476 °C. Chieruzzi et al. [45] reported a decrease of around 8 °C in the melting point of solar salt when doped with silica-alumina (SiO_2_-Al_2_O_3_) nanoparticles, and later also observed a 2 °C drop in the melting point of potassium carbonate when doped with silica nanoparticles (1 wt.%) as well [46]. Gavarrell et al. [47] reported a decrease of 2–3 °C in the melting point of a eutectic nitrate salt mixture doped with carbon nanotubes (CNT) compared to the pure salt. Studies on molten salt nanofluids using an in situ synthesis method have also reported anomalous changes (decreasing or increasing) in the onset and melting temperatures [18,19,20,21], even ranging up to 19 °C in some samples as reported by Huang et al. [20]. It is possible that the in situ production method might result in a significant change in the melting point compared to conventional nanofluid synthesis methods because the precursor materials are added in relatively higher concentrations and some traces could be present in the salt even after the thermal decomposition process is complete. The existence of impurities or small variations in the composition ratios of a mixture can noticeably alter its melting point [48]. It should be noted that the decrease of the melting point of molten salts is a favorable effect of adding nanoparticles, especially in the context of using molten salts as heat transfer fluids in concentrated solar power (CSP) plants or other applications where salt freezing can be a significant issue [49].

### 3.3. Material Characterization

**Figure 5:**
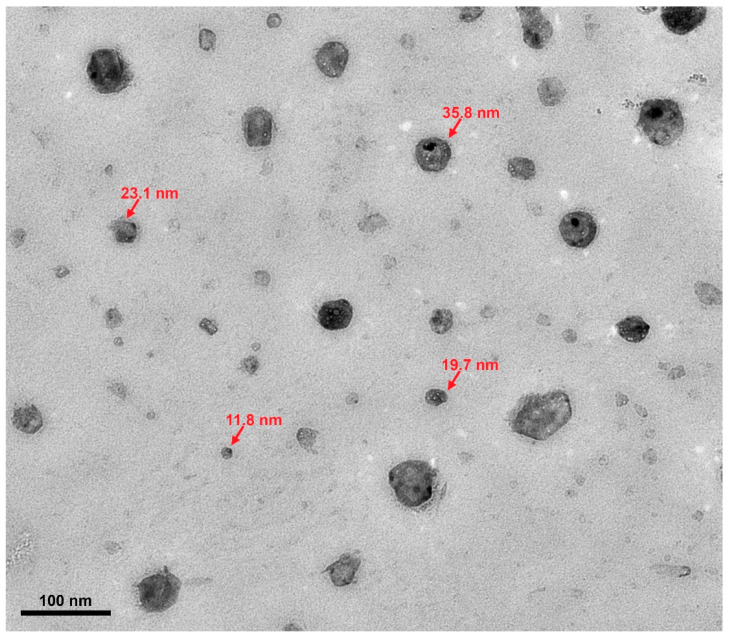
A TEM image of the in situ formed nanoparticles after separated from the salt.

To confirm the formation of nanometer-sized alumina particles, the nanoparticles were extracted from the salts by dissolving in distilled water on a TEM sample holder and characterized under a transmission electron microscope (TEM). The dissolving step was necessary to observe the nanoparticles; otherwise, nanoparticles are enclosed by salt eutectics and each specimen is too thick for the beam to transmit through. The existence of nanometer-sized alumina was confirmed as shown in the TEM micrograph in Figure 5. The image shows that the nanoparticles are spherical but not very uniform, the sizes of 24 nanoparticles were measured using ImageJ analysis software (1.52v, developed by National Institute of Health, Bethesda, MD, USA) to estimate the size distribution. The average size and the standard deviation of the nanoparticles were found to be 28.7 nm and 11.7 nm, respectively.

**Figure 6:**
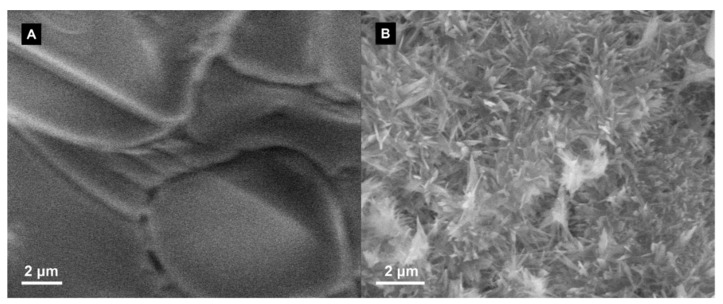
SEM micrographs. (**A**) The pure salt eutectic. (**B**) The salt nanofluid shows significant change in the microstructure of the salt.

SEM images of the pure salt eutectic and the salt nanofluid are shown in Figure 6. The pure salt eutectic did not show any distinct structure. On the other hand, the SEM image of the salt nanofluid shows a significant change in the microstructure. The change in the microstructure of molten salt nanofluid has been previously reported as one of the possible mechanisms behind the increase of heat capacity [8]. There have been several attempts in the literature to explain the enhanced heat capacity. They include the enhancement in heat capacity of nanometer-sized particles [50], interfacial thermal resistance and liquid layering on the nanoparticles’ surfaces [10,51], ion exchange between the salt ions and the nanoparticle [52], and the crystallization and growth of dendritic salt structures on the surface of the nanoparticles [12,32,37].

**Figure 7:**
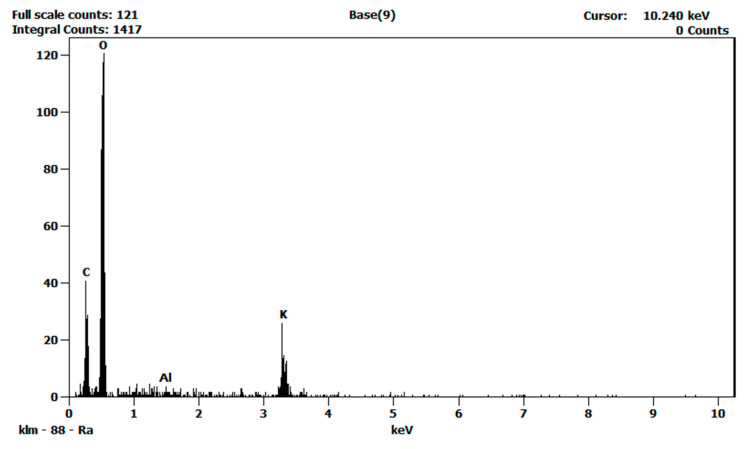
EDS spectra of the pure salt eutectic. Al peak is not shown.

**Figure 8:**
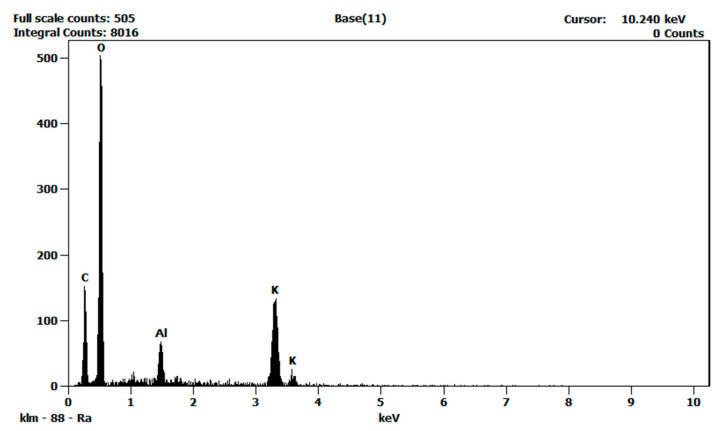
EDS spectra of the salt nanofluid. The peak corresponding to aluminum shows the presence of alumina nanoparticles.

Moreover, energy dispersive X-ray analyses (EDS) were performed to characterize the atomic compositions of the pure salt eutectic and the nanofluid. Figure 7 shows the resulting spectra of the EDS analysis performed on the pure salt eutectic and Figure 8 shows that of the salt nanofluid. The EDS analyses confirmed the presence of alumina (Al_2_O_3_) nanoparticles in the nanofluid.

## 4. Conclusions

In this study, a molten salt-based nanofluid was successfully synthesized via in-situ production of dispersed alumina nanoparticles in a binary carbonate salt eutectic (Li_2_CO_3_-K_2_CO_3_) using aluminum nitrate nonahydrate as a precursor. The nanoparticles were examined using TEM and were found to be nearly spherical in shape with an average diameter of 28.7 nm. The specific heat capacity of the nanofluid had an average value of 1.95 kJ/kg·°C compared to 1.61 kJ/kg·°C for the pure salt. The average enhancement in heat capacity was found to be about 21%. SEM micrographs of the nanofluid also showed significant changes in the microstructure of the salt, which could be linked to the enhancement of heat capacity.

This work demonstrates that the in situ nanofluid synthesis method is effective in producing molten salt nanofluids with comparable enhancement in heat capacity values to previously reported results on similar systems prepared using a complicated and time-consuming liquid solution method [53,54]. This method is expected to reduce the material/manufacturing costs of molten salt nanofluids significantly, making it a good candidate technique for a large-scale production of molten salt nanofluids for various applications including concentrated solar power (CSP).

## Figures and Tables

**Table 1:** A summary of the thermal decomposition process.

Reaction	Temperature		%Weight	
	Ref. [39]	Measured	Theoretical	Measured
$2Al{\left(NO_{3}\right)}_{3}\cdot 9H_{2}O\to 2Al\left(NO_{3}\right)+9H_{2}O$	130 °C	132 °C	97.01%	97.49% (132 °C)
$2Al\left(NO_{3}\right)\to Al_{2}O_{3}+3NO_{2}+3NO+3O_{2}$	200 °C	205 °C	94.02%	95.17% (205 °C) 93.79% (540 °C)

**Table 2:** A summary of the heat capacity and the melting point measurements of the pure salt (Li_2_CO_3_-K_2_CO_3_). All C_p_ values are reported at 550 °C.

Samples	Heat Capacity (STDEV) (kJ/kg·°C)	Melting Point (STDEV) (°C)
1	1.63 (0.09)	486 (0.02)
2	1.57 (0.06)	484 (0.60)
3	1.62 (0.06)	485 (0.96)
Average	1.61	485
Standard deviation	0.065	1.22
Measurement uncertainty (%)	3.31	0.21

**Table 3:** A summary of the heat capacity and the melting point measurements of the salt nanofluid (Li_2_CO_3_-K_2_CO_3_ with alumina nanoparticles). All C_p_ values are reported at 550 °C.

Samples	Heat Capacity (STDEV) (kJ/kg·°C)	Melting Point (STDEV) (°C)
1	2.29 (0)	476 (0.9)
2	1.9 (0.02)	475 (0.01)
3	1.68 (0.08)	474 (0.12)
4	2.05 (0.09)	474 (0.1)
5	1.78 (0.09)	473 (0.05)
6	1.94 (0.11)	473 (0.22)
7	1.87 (0.14)	473 (0.23)
8	1.84 (0.11)	473 (0.23)
9	1.81 (0.05)	471 (2.3)
10	2.14 (0.02)	475 (0.1)
11	2.09 (0.14)	476 (0.02)
12	2.06 (0.02)	475 (0.07)
Average	1.95	474
Standard deviation	0.18	1.48
Measurement uncertainty (%)	3.18	0.11
